# Diffusion Tensor Imaging Profiles Can Distinguish Diffusivity and Neural Properties of White Matter Injury in Hydrocephalus vs. Non-hydrocephalus Using a Strategy of a Periodic Table of DTI Elements

**DOI:** 10.3389/fneur.2022.868026

**Published:** 2022-07-06

**Authors:** Nicole C. Keong, Christine Lock, Shereen Soon, Aditya Tri Hernowo, Zofia Czosnyka, Marek Czosnyka, John D. Pickard, Vairavan Narayanan

**Affiliations:** ^1^Department of Neurosurgery, National Neuroscience Institute, Singapore, Singapore; ^2^Duke-NUS Medical School, Singapore, Singapore; ^3^Department of Surgery, Faculty of Medicine, University of Malaya, Kuala Lumpur, Malaysia; ^4^Neurosurgical Division, Department of Clinical Neurosciences, University of Cambridge, Cambridge, United Kingdom

**Keywords:** normal pressure hydrocephalus (NPH), diffusion tensor imaging (DTI), white matter, traumatic brain injury (TBI), Alzheimer's disease, injury properties

## Abstract

**Background:**

The aim of this study was to create a simplistic taxonomy to improve transparency and consistency in, and reduce complexity of, interpreting diffusion tensor imaging (DTI) profiles in white matter disruption. Using a novel strategy of a periodic table of DTI elements, we examined if DTI profiles could demonstrate neural properties of disruption sufficient to characterize white matter changes specific for hydrocephalus vs. non-hydrocephalus, and to distinguish between cohorts of neural injury by their differing potential for reversibility.

**Methods:**

DTI datasets from three clinical cohorts representing pathological milestones from reversible to irreversible brain injury were compared to those of healthy controls at baseline, over time and with interventions. The final dataset comprised patients vs. controls in the following groupings: mild traumatic brain injury (mTBI), *n* = 24 vs. 27, normal pressure hydrocephalus (NPH), *n* = 16 vs. 9 and Alzheimer's disease (AD), *n* = 27 vs. 47. We generated DTI profiles from fractional anisotropy (FA) and mean, axial and radial diffusivity measures (MD, L1 and L2 and 3 respectively), and constructed an algorithm to map changes consistently to a periodic table of elements, which fully described their diffusivity and neural properties.

**Results:**

Mapping tissue signatures to a periodic table of DTI elements rapidly characterized cohorts by their differing patterns of injury. At baseline, patients with mTBI displayed the most preserved tracts. In NPH, the magnitude of changes was dependent on “familial” DTI neuroanatomy, i.e., potential for neural distortion from risk of ventriculomegaly. With time, patients with Alzheimer's disease were significantly different to controls across multiple measures. By contrast, patients with mTBI showed both loss of integrity and pathophysiological processes of neural repair. In NPH, some patterns of injury, such as “stretch/compression” and “compression” were more reversible following intervention than others; these neural profile properties suggested “microstructural resilience” to injury.

**Conclusion:**

Using the novel strategy of a periodic table of DTI elements, our study has demonstrated it is possible to distinguish between different cohorts along the spectrum of brain injury by describing neural profile properties of white matter disruption. Further work to contribute datasets of disease toward this proposed taxonomic framework would enhance the translatability of DTI profiles to the clinical-research interface.

## Introduction

Restoration of brain functions following injury requires an understanding of the resilience of neural tissue against pathological insults. Currently, we assess white matter tracts for microstructural damage but do not describe them in terms of their potential reversibility. Participants in longitudinal cohort studies and clinical trials undergo baseline imaging to document their structural integrity and functional connectivity. However, they may have white matter injury patterns with differing capacity for neural repair or recovery. Such patients may not exhibit equivalent responses to interventions, including drug therapies or application of novel neuroprostheses. Yet, there is no standard framework to describe cohorts by their differing white matter injuries. This gap in knowledge and such differential potential of patient cohorts are likely to have been major contributors to the failures of promising drug interventions in large-scale clinical trials across acute brain injury and neurodegenerative disease.

There is an urgent need to develop more precise imaging biomarkers for correlation with assessments of intervention. In addition, our lack of understanding of how to describe the potential responsiveness of white matter injury has implications for assessing the risks vs. benefits for specific interventions in high-risk groups, such as the elderly population. The emerging field of aging neurosurgery represents such an evolving challenge. Despite the growth of the cohort of patients above 65 years presenting for surgical intervention, a cohesive approach toward a strategy for geriatric surgical needs is lacking. Elderly patients are considered high risk and yet, demonstrate potential for recovery. Specific conditions, such as chronic subdural haematoma, spinal degenerative myeloradiculopathy and hydrocephalus, have excellent results with surgical intervention (1–3). Good outcomes may be further supported with the use of risk stratification, such as frailty and comorbidity scoring. However, continuing neurological improvement may be dependent on the capacity of white matter injury for microstructural resilience. A key gap in our approach is the paucity of tools to describe the potential for neural injury to recover from pathological insults, with or without intervention. In this regard, it may be helpful to study Normal pressure hydrocephalus (NPH) as a model of reversible brain injury.

Classically described by Adams et al. (4) as a triad of gait disturbance, cognitive decline and urinary incontinence, NPH attracts up to 96% chance subjective improvement and 83% chance improvement on timed walk test at 6 months (3). Although increasing age is not a prognostic factor, various factors limit consistently good outcomes across patient groups. Challenges include developing more reliable non-invasive imaging methods for higher diagnostic certainty, with a greater capacity for predicting shunt-responsiveness (5). Symptoms attributed to the NPH triad also appear in other hydrocephalic conditions, in a spectrum from compensated to subacute obstructive forms, for which differing surgical strategies may be optimal (6). Whilst NPH is characterized by its distinctive gross ventriculomegaly, degree of ventricular dilatation is neither predictive of clinical nor functional improvement. In some cases, but not all, NPH is associated with defective cerebrospinal fluid (CSF) circulation and pressure-volume compensation. In such Classic NPH subtypes, interrogation of cerebral hydrodynamics via a CSF infusion test can be helpful in predicting improvement after shunting (7, 8). However, a further complication exists in the form of overlay with comorbid diseases. Distinctive radiological features in NPH, such as ventriculomegaly, may be common to hydrocephalus, brain atrophy as well as neurodegeneration. Neurodegenerative disorders, including Alzheimer's disease (AD), frequently co-present in the cohort with NPH symptoms. In our previous work, we have described a subtype we term “Complex NPH.” This challenging cohort presents with overlay from multiple co-morbidities co-existing, particularly vascular risk burden and neurodegenerative diseases; patients still show capacity for CSF responsiveness, but testing is difficult (9). Such subtypes of Complex NPH, or patients with frailty, may benefit from screening *in vivo* to characterize the reversibility potential of their neural injury prior to invasive CSF testing.

The study of NPH is confounded by multiple theories of pathogenesis, yet unresolved. However, many major hypotheses streams, such as tissue distortion and vascular ischaemia, converge in white matter dysfunction (5). Shunt-responsive NPH patients may be demonstrating a form of ‘microstructural resilience'; their white matter exhibits potential to recover from injury. Diffusion tensor imaging (DTI), has been established as a robust and reliable way of interrogating tissue microstructure, with both the capacity to document patterns of white matter injury in NPH, as well as reversibility post-shunting (10). Whilst this technique is well-accepted in conditions such as stroke, tumors and traumatic brain injury (11–15), and readily available in academic clinical centers, there are technical challenges to overcome. DTI metrics are “semi-quantitative”; dependent on (i) machine factors–site/scanner-specific, b values, number of diffusion directions, (ii) biological confounders–multiple pathophysiological processes co-existing, crossing fibers, ongoing insults/ recovery across timepoints and (iii) interpretations–post-processing algorithms and assumptions, handling of DTI conflicts. In response, there has been increasing development of higher scanning specifications and advanced imaging processing methodologies. However, such strategies reduce its attractiveness as a rapid, first-line screening tool at the clinical-research interface.

**Figure 1:**
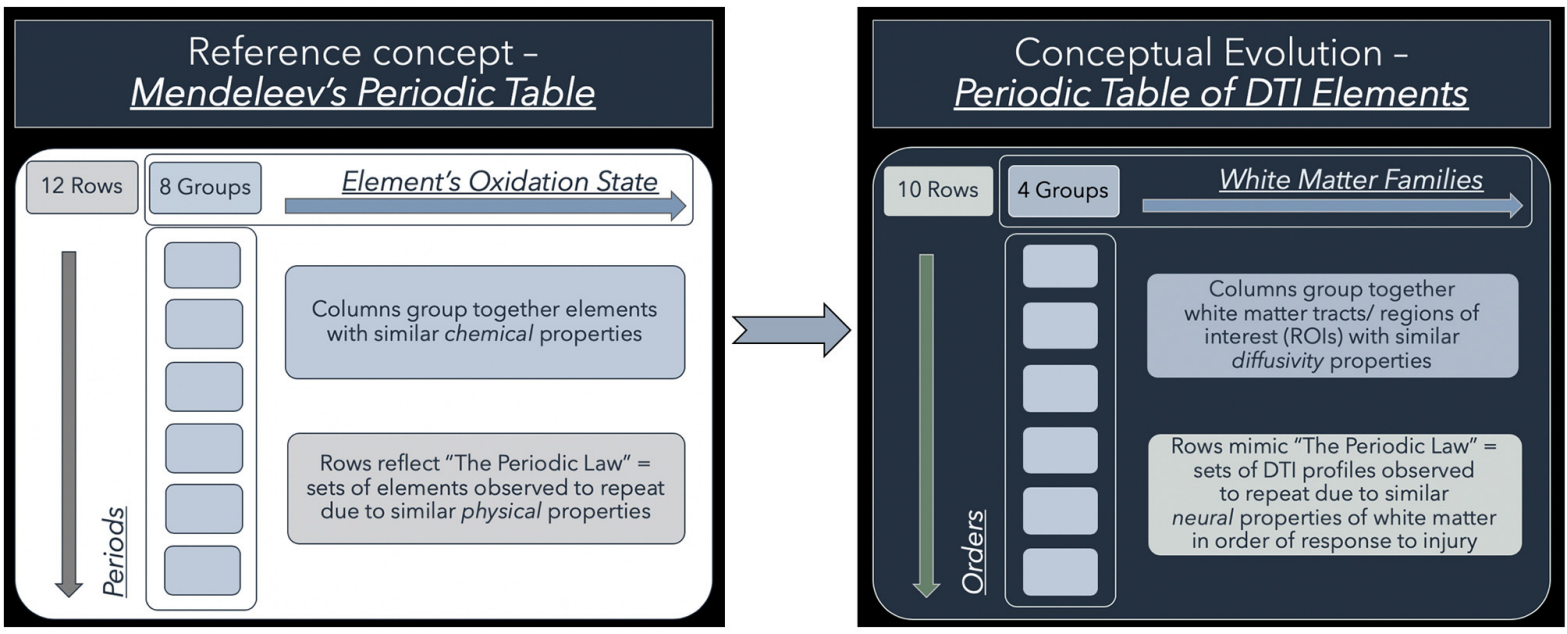
Conceptual evolution from Mendeleev's Periodic Table to the current work. The modern periodic table in chemistry is based on atomic number. This differs from Mendeleev's original concept, which arranged the elements according to their similar chemical and physical properties. Mendeleev sorted the elements largely by atomic mass but where there were tensions in the order, he prioritized groups by their shared properties. Similarly, we have used shared “*diffusivity”* properties to group white matter tracts/ regions of interest (ROIs) into columns. This grouping into “*White matter families”* reflects their DTI neuroanatomy, changes in diffusivity profiles due to their differential risk from progressive ventriculomegaly. Mimicking the concept of periods, we have arranged sets of DTI profiles observed to repeat due to similar “*neural”* properties. These rows, which we have termed “*Orders*,” reflect commonly occurring DTI profiles seen in white matter in response to injury. Note that the Order from I to X here reflects a predicted trend from reversible to irreversible injury. This is arbitrarily defined and could equally work in reverse order as long as the sequence is maintained.

To address this conundrum, we have previously shown the utility of simplistic ROI-based DTI profiles in an at-risk model of white matter injury in NPH (10). DTI profiles are a methodology of distilling the complexity of changes, occurring across multiple DTI measures concurrently, into their most simplistic, graphical forms (9). DTI profiles display both the magnitude and direction of predominant changes to describe patterns of white matter injury in terms of their diffusion morphology. Here, we present a further contribution toward this concept. We found that, despite conflicts in DTI measures, we could observe recurring properties in DTI profiles of common patterns of white matter injury seen across cohorts. Inspired by the discussions surrounding the recent 150th anniversary of Mendeleev's periodic table in chemistry, we examined whether our observations would benefit from being organized into such an array. Mendeleev arranged his table by atomic weight, according to recurring chemical and physical properties of elements and confirmed the validity of the Periodic Law (16, 17). We believed it was possible to evolve from this concept to the notion of arranging recurring DTI profiles in a similar way (Figure 1). We therefore proposed a novel arrangement of white matter injury by their familial DTI neuroanatomy, rather than by functional considerations. As we based our table on the periodicity of “diffusivity” and “neural” properties of white matter in response to injury, we termed this a “*periodic table of DTI elements*.” The aims of this study were to (1) examine if DTI profiles could distinguish white matter injury in hydrocephalus vs. non-hydrocephalus and if so, (ii) create a simplified taxonomy of DTI interpretation by using the strategy of a “*periodic table of DTI elements*.”

## Methods

We examined DTI profiles from a cohort of Classic NPH (ClNPH) patients, the purer form of shunt-responsive NPH, and compared them to patients with mild traumatic brain injury (mTBI; who exhibit both trajectories of recovery and persisting neurological deficits) and patients with Alzheimer's disease (AD; an exemplar cohort of irreversible brain injury due to progressive neurodegeneration). The utility of DTI examination has been shown in all three conditions; interpretations of white matter injury patterns are known (10–12, 18–23).

DTI metrics for the various clinical cohorts mTBI, ClNPH and AD–were obtained from international datasets for which methodology is well-established. Cohort demographics are described in Table 1. The mTBI cohort from the University of Malaya comprised 62 patients who presented with mTBI and 27 age-matched healthy controls (21 males and 6 females; mean age 29.0 years). In this analysis, we included only the 24 mTBI patients (20 males and 3 females; mean age 28.6 years) who had baseline DTI measures and 6-month follow-up data available. This dataset has been expanded from the previously published series (61 patients and 19 controls) (24), in which DTI profiles were not examined. Pre- and post-operative DTI measures for the ClNPH group, which included 16 patients with NPH (10 males and 6 females; mean age 74.7 years) and 9 healthy controls (4 males and 5 females; mean age 70.0 years), were derived from a cohort we have previously published from the University of Cambridge (10). We have previously demonstrated the use of DTI profiles in the ClNPH group (9, 10); we have expanded on the published data by including 6-month post-operative data in our analysis. Data for the AD cohort were obtained from the Alzheimer's Disease Neuroimaging Initiative (ADNI) database (adni.loni.usc.edu) and included 27 patients with AD baseline and 12-month follow-up DTI measures available (20 males and 7 females; mean age 75.6 years) and 47 healthy controls (22 males and 25 females; mean age 72.9 years). Using data expanded from published series was critical to testing the novel strategy of the periodic table of DTI elements as interpretation of injury patterns had already been established.

**Table 1:** Demographics for cohorts.

	**Mild traumatic brain injury cohort**		**Normal pressure hydrocephalus cohort**		**Alzheimer's disease cohort**	
	**Healthy controls**	**mTBI**	Healthy controls*	**NPH**	**Healthy controls**	**AD**
n	27	24	9	16	47	27
Age (Mean ± SD)	29.0 ± 6.76	28.6 ± 9.34	70.0	74.7 ± 5.88	72.9 ± 6.04	75.6 ± 8.52
Age (Range)	18–49	18–50	N.A.	60–84	59.9–89.1	61.8–90.4
Sex (% male)	77.8	83.3	44.4	62.5	46.8	74.1

*Demographics reported are from published data (10). N.A., data not available.

Approval for individual studies was obtained from local or institutional ethics committees. Written informed consent was obtained from all subjects or legal representatives, if appropriate, as required by local ethics committees.

### DTI Acquisition

The mTBI cohort from the University of Malaya was imaged on a 3T MRI scanner (Signa HDx; General Electric, Harvey, IL) using an eight-channel head coil (24). The DTI sequence was obtained using these parameters: TR = 13,000 ms; TE = 81.2 ms; FOV = 24 mm; matrix = 128 x 128; slice thickness = 3.0 mm; 32 directions; diffusion weighted factor, b = 700 s/mm^2^; and image scan time of 7 min 22 s.

MRI for the NPH cohort was performed on a 3T Siemens Tim Trio using a 12 channel head matrix radio frequency receive coil (10). The DTI sequence was acquired by using a spin echo diffusion weighted echo planar imaging sequence with the following parameters: TR/TE, 8,300 ms/98 ms, matrix dimensions 96 x 96, FOV 192 x 192, slice thickness 2 mm giving a voxel size of 2 x 2 x 2 mm. Diffusion weighted images were acquired in 12 non-collinear directions each at 5 b-values of 350, 650, 1,000, 1,300 and 1,600 s/mm^2^, along with 4 b-0 images.

The AD cohort from the ADNI were scanned on 3T GE Medical Systems scanners at 14 acquisition sites in North America (25). Diffusion weighted images were acquired with the following parameters: 256 × 256 matrix; voxel size: 2.7 × 2.7 × 2.7 mm^3^; TR = 9,000 ms; scan time = 9 min. Forty-six separate images were acquired for each DTI scan: 5 T2-weighted images with no diffusion sensitization (b0 images) and 41 diffusion-weighted images (b = 1,000 s/mm^2^).

### Pre-processing and ROI Analysis

Pre-processing, image registration, and analysis for the mTBI dataset was carried out with FSL and AFNI software packages. Initial preprocessing involved corrections for head movement and eddy currents, brain tissue extraction, and fitting of the diffusion tensor model. The FSL tool fnirt was used for non-linear special registration of each subject to the FMRIB58_FA standard-space image. Predefined ROIs for each individual subject were mapped with the AFNI 3dROIstats tool and median FA, MD, L1, and L2 and 3 values obtained for each tract (24).

In the NPH dataset, the FDT diffusion toolbox in FSL analysis tools was used for eddy current correction and DTI pre- and post-processing. Co-registration of the structural 3D volumetric image and DTI images was performed using SPM5. MPRAGE images were re-sliced to match the DTI image space using the s0 volume (the b = 0 volume with optimal signal-to-noise ratio). A DTI-based white matter tract atlas was used for anatomical identification of key fiber bundles and ROIs were placed on the structural images and applied to co-registered DTI files (10).

ADNI DTI metrics used in this paper were the UCLA DTI ROI summary measures for ADNIGO and ADNI2. Pre-processing steps for head motion and eddy current correction and removal of non-brain tissue were done using FSL. T1-weighted anatomical images were aligned to a standard brain template; skull-stripped b0 images were aligned and then registered to their respective T1-weighted scans. A study-specific template was created, each individual subject's initial FA map elastically registered to the template, and resulting deformation fields applied to diffusivity maps to align them to the same coordinate space. The mean of all voxels from each of the regions of interest from the atlas were obtained (25).

### Generation of DTI Profiles

**Figure 2:**
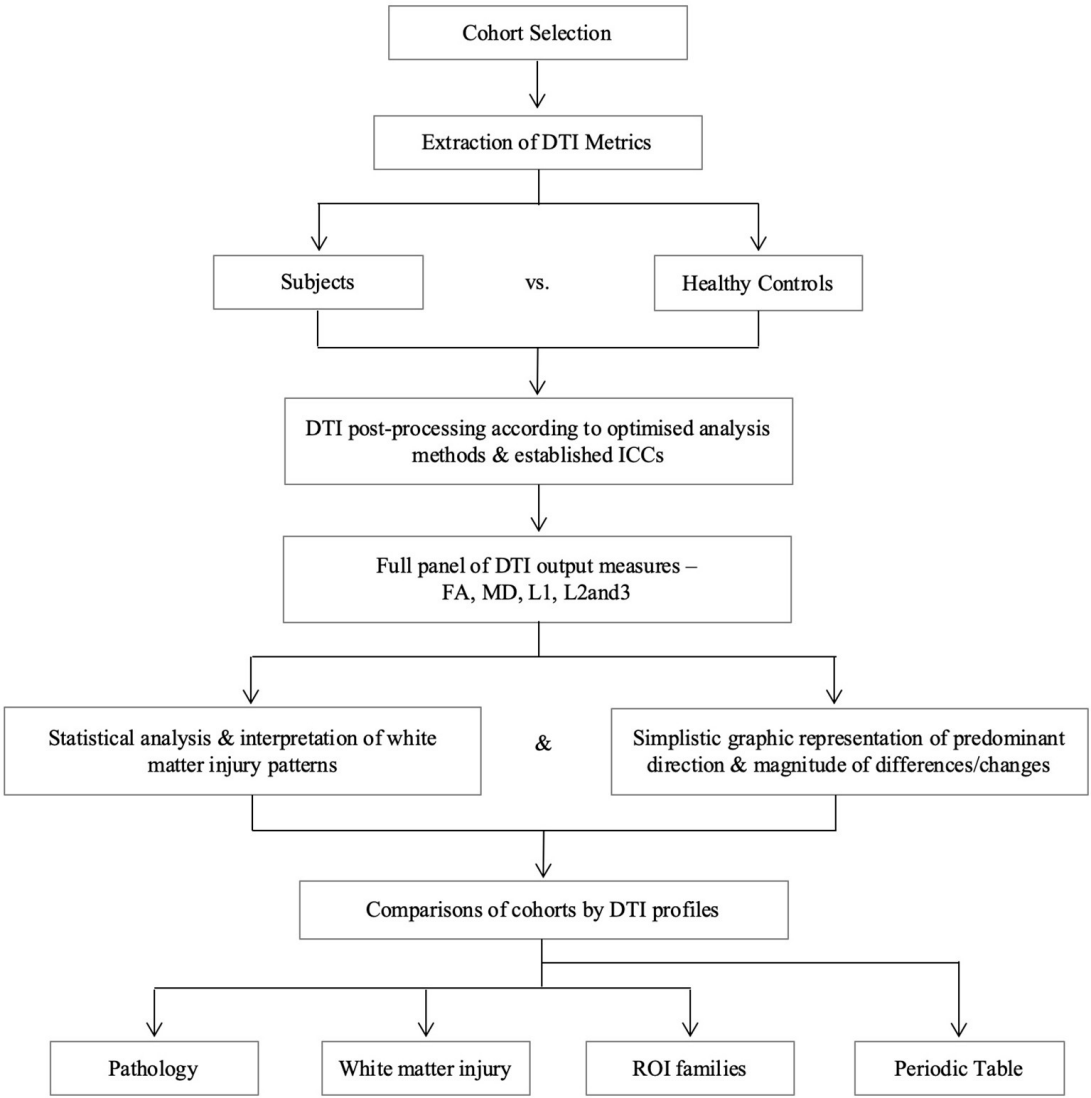
Overview of study methodology. We refer to axial and radial diffusivities by their eigenvalues for clarity (L1 and L2 and 3, respectively), to distinguish these measures from terms we use for pathological cohorts. DTI, diffusion tensor imaging; FA, fractional anisotropy; ICC, intraclass correlation coefficient; L1, axial diffusivity, L2 and 3, radial diffusivity; MD, mean diffusivity; ROI, region of interest.

**Figure 3:**
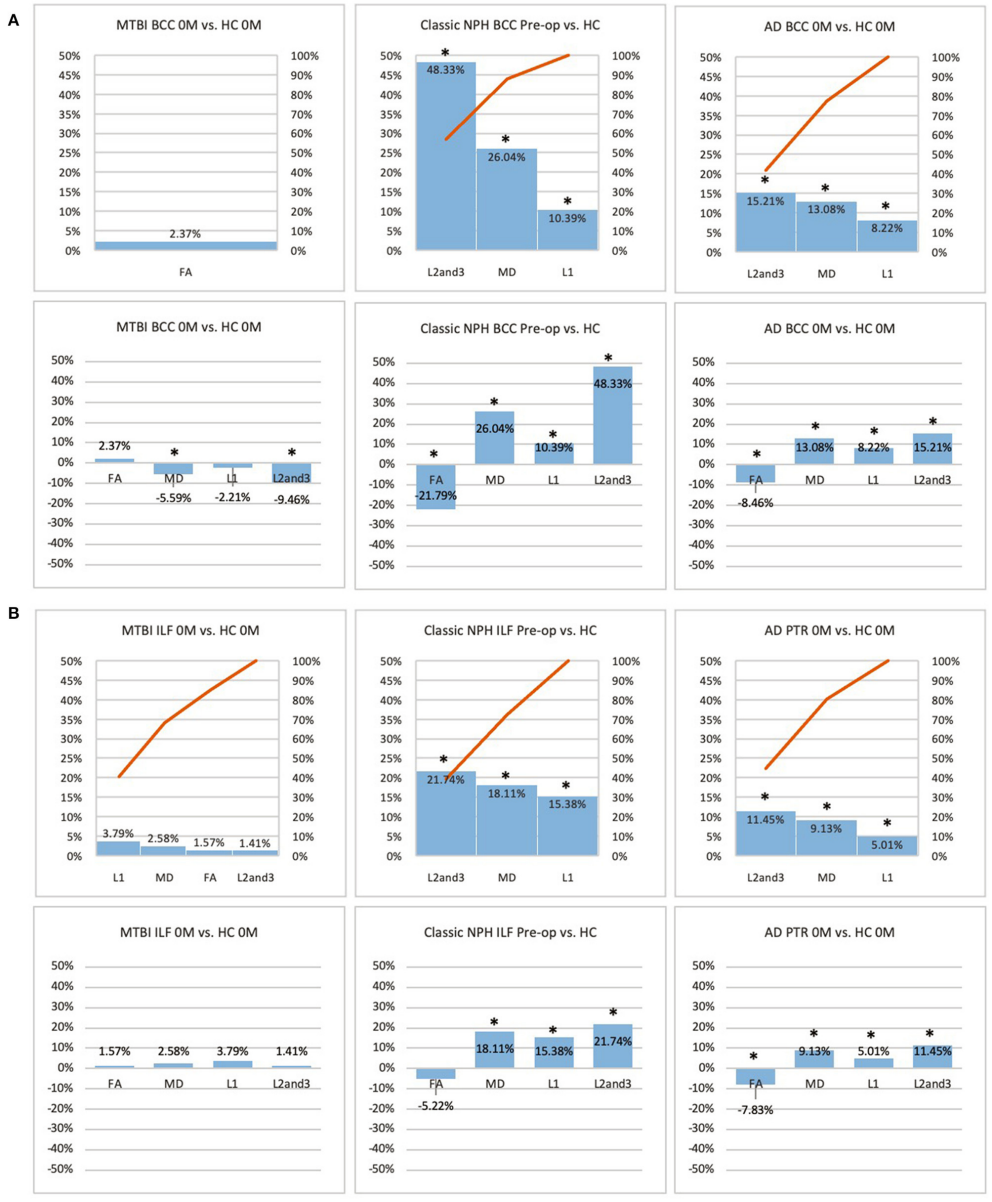
**(A)** Midline (BCC) and **(B)** lateral (ILF or PTR) ROI families at baseline. At baseline, percentage differences in DTI measures of clinical cohorts are compared against corresponding healthy controls, presented as Pareto graphs and bar charts. In Pareto graphs, positive values are automatically arranged sequentially, in order of highest to lowest magnitude of differences/changes. *Indicates a significant Difference between the clinical cohort and healthy controls at *p* < 0.05.

**Figure 4:**
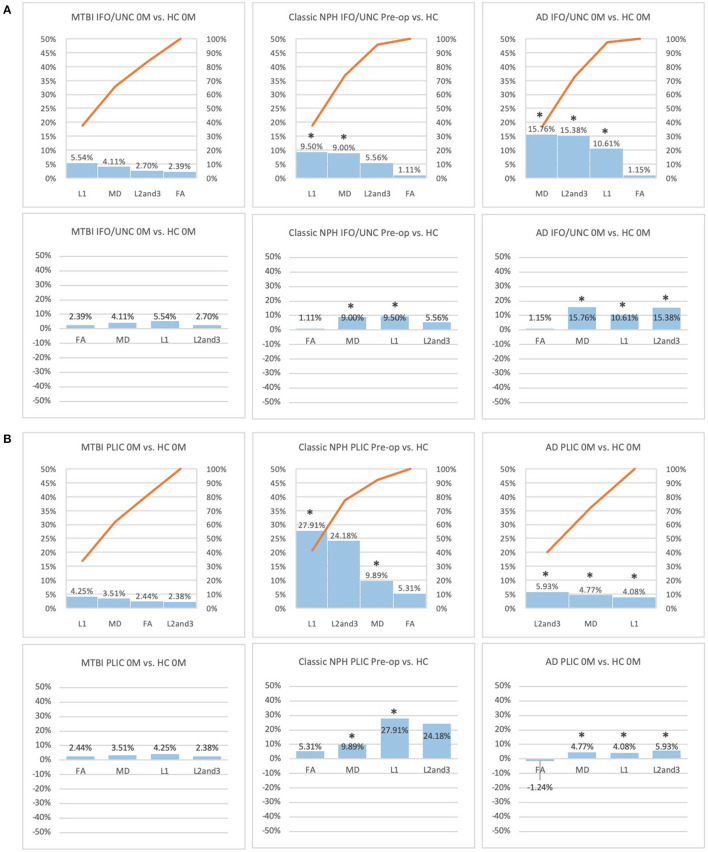
**(A)** Fronto-temporal (IFO/UNC) and **(B)** remote functional (PLIC) ROI families at baseline. At baseline, percentage differences in DTI measures of clinical cohorts are compared against corresponding healthy controls, presented as Pareto graphs and bar charts. In Pareto graphs, positive values are automatically arranged sequentially, in order of highest to lowest magnitude of differences/changes. * indicates a significant difference between the clinical cohort and healthy controls at *p* < 0.05.

**Figure 5:**
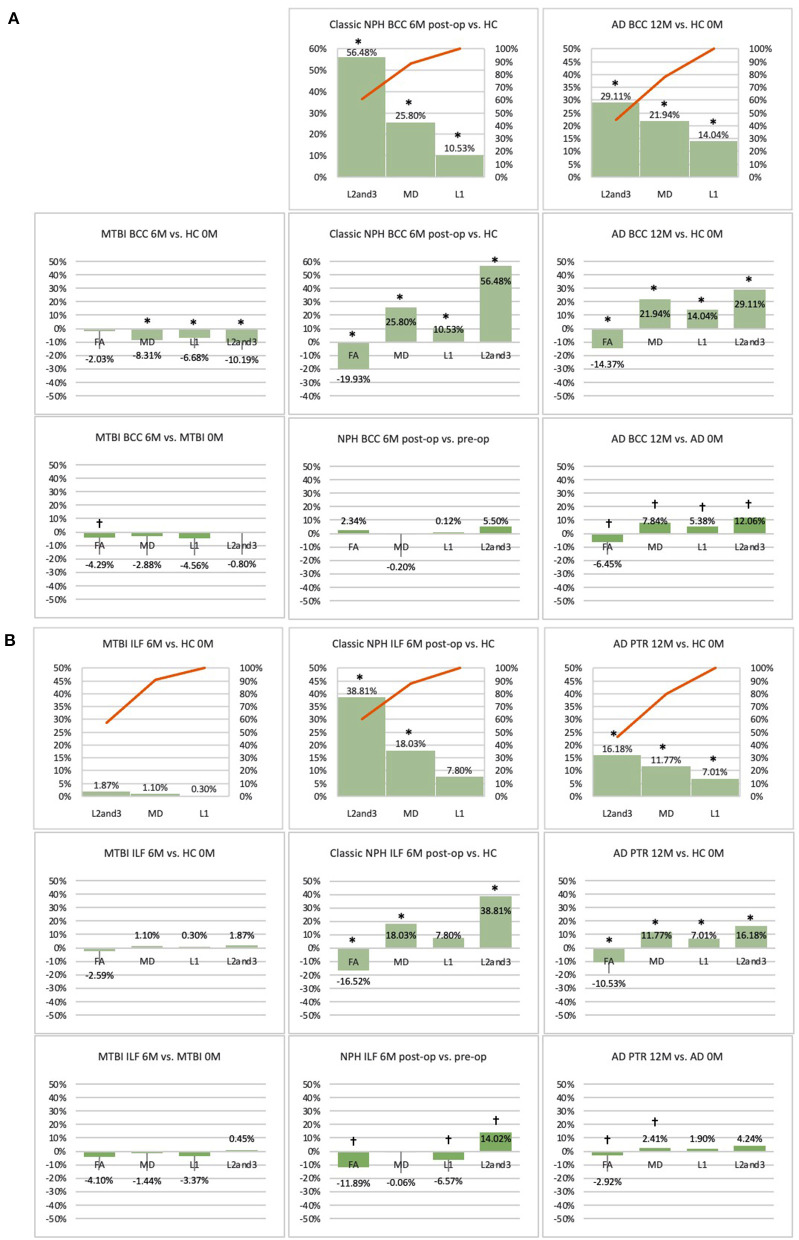
**(A)** Midline (BCC) and **(B)** lateral (ILF or PTR) ROI families at follow-up. At 6 or 12-month follow-up, percentage changes in DTI measures of clinical cohorts are compared against corresponding healthy controls at baseline, presented as Pareto graphs and bar charts. At this time point, patients are also compared to themselves at baseline, presented as bar charts. We observed that, in MTBI, the BCC ROI at 6-month follow-up demonstrated an unique profile (significant decreases in mean, axial and radial diffusivity measures in the context of a non-significant decrease in FA). We suggest this is possibly indicative of processes seen in the spectrum of neural repair. At this timepoint in NPH, we found changes driven by a disproportionate increase in radial diffusivity, a DTI profile reported in other subtypes of post-operative hydrocephalus. In AD at 12-month follow-up, we found global significant deterioration across mean, axial and radial diffusivity measures, consistent loss of integrity/atrophy. By contrast, we observed that, for the ILF or PTR ROI, there was preservation of white matter tracts in MTBI at 6-month follow-up. At this timepoint in NPH, we found changes indicative of improvement in compression (a significant increase in radial diffusivity but in the context of a decrease in axial diffusivity). In AD at 12-month follow-up, we observed significant worsening of white matter due to increase in loss of integrity/atrophy. *Indicates a significant Difference between the clinical cohort and healthy controls at *p* < 0.05. ^†^Indicates a significant Change in the clinical cohort across timepoints at *p* < 0.05.

**Figure 6:**
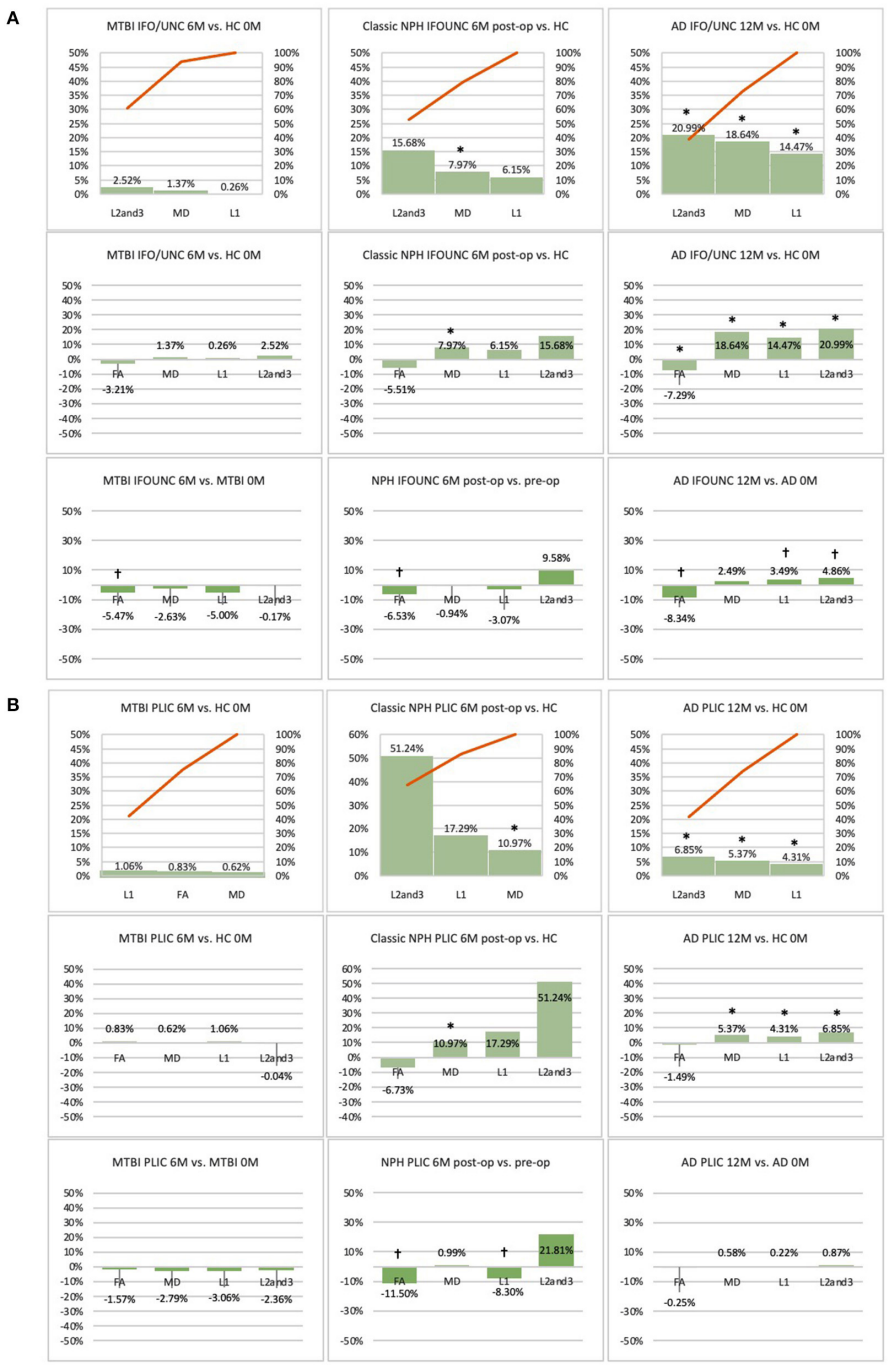
**(A)** Fronto-temporal (IFO/UNC) and **(B)** remote functional (PLIC) ROI families at follow-up. At 6 or 12-month follow-up, percentage changes in DTI measures of clinical cohorts are compared against corresponding healthy controls at baseline, presented as Pareto graphs and bar charts. At this time point, patients are also compared to themselves at baseline, presented as bar charts. We observed that, in MTBI, the IFO/UNC combination ROI at 6-month follow-up demonstrated a pattern of distortion/oedema (predominant increase in radial diffusivity with a significant decrease in FA), whereas in NPH, we found changes consistent with non-significant improvement in distortion of the tract (increase in radial diffusivity but in the context of decreases in axial and mean diffusivities). In AD at 12-month follow-up, we found global deterioration across all diffusivity measures (with a significant decrease in FA) consistent with progressive neurodegeneration. By contrast, we observed that, for the PLIC ROI, there was preservation of white matter tracts in MTBI at 6-month follow-up and in NPH, improvement in compression (increase in radial diffusivity but in the context of a significant decrease in axial diffusivity). In AD at 12-month follow-up, we observed non-significant worsening of white matter due to likely neurodegeneration. *Indicates a significant Difference between the clinical cohort and healthy controls at *p* < 0.05. ^†^Indicates a significant Change in the clinical cohort across timepoints at *p* < 0.05.

DTI region of interest (ROI) values for fractional anisotropy (FA), mean diffusivity (MD), axial diffusivity (L1), and radial diffusivity (L2 and 3) obtained using established methodology were used in this analysis (10, 24, 25). Axial and radial diffusivities are referred to by their eigenvalues (L1 and L2 and 3, respectively) to avoid confusion with acronyms for pathology, e.g., AD for Alzheimer's Disease. Previous work in the NPH study confirmed no significant differences in right- and left-sided ROIs, therefore right-sided tracts were used for this study to avoid over-averaging across multiple ROIs and within groups. However, as midline tracts were acquired as single ROIs for the NPH and mTBI groups, the averaged value of right and left body and genu of the corpus callosum in the AD group was used for comparison. We generated DTI profiles as per the workflow in Figure 2. Here, we preferred Pareto graphs; this graphical format automatically displays positive DTI measures in order of decreasing magnitude of changes, thereby allowing concurrent examination of both predominant direction and magnitude of differences. To reflect smaller, and usually negative changes in FA, we also correlated findings seen on the Pareto graphs with bar charts and standard statistical analyses (Figures 3–6, Tables 2, 3).

**Table 2:** Comparison of example DTI region of interest values between clinical cohorts and healthy controls.

**DTI Regions of Interest**	**Example ROI**	**Cohort**	**Time- point**	***n***	**FA**	% dif ference*	*p*- value*	**MD (10^**−4**^ mm^**2**^/s)**	% dif ference*	*p*- value*	**L1 (10^**−4**^ mm^**2**^/s)**	% dif ference*	*p*- value*	**L2 and3 (10^**−4**^ mm^**2**^/s)**	% dif ference*	*p*- value*
Midline	BCC	HC	0 M	27	0.508 ± 0.032			9.704 ± 0.76			15.626 ± 0.98			6.742 ± 0.75		
right-left tracts		mTBI	0 M	24	0.520 ± 0.065	+2.4	0.418	9.162 ± 1.07	−2.6	**0.040**	15.280 ± 2.21	−1.1	0.465	6.104 ± 0.70	−4.2	**0.003**
adjacent to		mTBI	6 M	24	0.498 ± 0.057	−2.0	0.440	8.898 ± 0.32	−8.3	**<0.001**	14.583 ± 0.87	−6.7	**<0.001**	6.055 ± 0.45	−10.2	**<0.001**
ventricles		HC	0 M	9	0.612 ± 0.090			7.416 ± 1.08			13.072 ± 1.19			4.589 ± 1.29		
e.g., BCC		NPH Pre-op	0 M	16	0.478 ± 0.097	−21.8	**0.003**	9.348 ± 1.45	+26.0	**0.002**	14.430 ± 1.50	+10.4	**0.029**	6.807 ± 1.67	+48.3	**0.002**
GCC		NPH Post-op	6 M	16	0.490 ± 0.104	−19.9	**0.007**	9.329 ± 1.26	+25.8	**0.001**	14.448 ± 1.22	+10.5	**0.012**	7.181 ± 2.30	+56.5	**0.005**
		HC	0 M	47	0.387 ± 0.048			12.560 ± 1.18			17.958 ± 1.14			9.862 ± 1.26		
		AD	0 M	27	0.355 ± 0.053	−7.7	**0.008**	14.202 ± 1.58	+11.3	**<0.001**	19.434 ± 1.38	+6.6	**<0.001**	11.363 ± 1.83	+12.1	**0.001**
		AD	12 M	27	0.332 ± 0.075	−14.4	**<0.001**	15.315 ± 3.30	+21.9	**<0.001**	20.480 ± 2.65	+14.0	**<0.001**	12.733 ± 3.65	+29.1	**<0.001**
Lateral to	ILF/PTR	HC	0 M	27	0.416 ± 0.052			8.728 ± 0.41			12.842 ± 0.76			6.671 ± 0.51		
ventricles (long		mTBI	0 M	24	0.423 ± 0.077	+1.0	0.721	8.953 ± 1.08	+2.6	0.320	13.329 ± 2.77	+3.3	0.385	6.765 ± 0.45	+2.0	0.490
or short)		mTBI	6 M	24	0.405 ± 0.051	−2.6	0.457	8.824 ± 0.32	+1.1	0.361	12.880 ± 0.81	+0.3	0.863	6.796 ± 0.41	+1.9	0.344
anterior-		HC	0 M	9	0.575 ± 0.070			6.610 ± 1.14			11.302 ± 1.69			4.264 ± 0.95		
posterior		NPH Pre-op	0 M	16	0.545 ± 0.051	−5.2	0.233	7.807 ± 0.69	+18.1	**0.003**	13.040 ± 1.38	+15.4	**0.010**	5.191 ± 0.55	+21.7	**0.005**
e.g. ILF, ATR		NPH Post-op	6 M	16	0.480 ± 0.074	−16.5	**0.005**	7.802 ± 0.62	+18.0	**0.002**	12.184 ± 1.20	+7.8	0.141	5.919 ± 1.24	+38.8	**0.002**
tracts, PTR		HC	0 M	47	0.385 ± 0.033			9.212 ± 0.87			13.172 ± 0.92			7.228 ± 0.88		
CGC		AD	0 M	27	0.355 ± 0.028	−5.1	**<0.001**	10.054 ± 0.86	+6.8	**<0.001**	13.832 ± 0.97	+3.7	**0.005**	8.055 ± 0.98	+7.8	**<0.001**
		AD	12 M	27	0.345 ± 0.031	−10.5	**<0.001**	10.296 ± 1.08	+11.8	**<0.001**	14.095 ± 1.10	+7.1	**<0.001**	8.397 ± 1.09	+16.2	**<0.001**
Fronto-	IFO/ UNC	HC	0 M	27	0.434 ± 0.035			9.116 ± 0.32			13.714 ± 0.49			6.816 ± 0.40		
temporal		mTBI	0 M	24	0.444 ± 0.056	+1.4	0.428	9.491 ± 1.09	+2.3	0.095	14.473 ± 2.41	+3.0	0.116	7.000 ± 0.56	+1.5	0.181
multi-		mTBI	6 M	24	0.420 ± 0.034	−3.2	0.160	9.241 ± 0.34	+1.4	0.180	13.749 ± 0.53	+0.3	0.804	6.988 ± 0.41	+2.5	0.139
directional		HC	0 M	9	0.399 ± 0.048			6.550 ± 0.38			9.499 ± 0.57			5.075 ± 0.43		
tracts		NPH Pre-op	0 M	16	0.403 ± 0.059	+1.1	0.852	7.140 ± 0.42	+9.0	**0.002**	10.402 ± 0.75	+9.5	**0.005**	5.358 ± 0.74	+5.6	0.306
e.g. IFO, UNC		NPH Post-op	6 M	16	0.377 ± 0.054	−5.5	0.319	7.072 ± 0.47	+8.0	**0.009**	10.083 ± 0.73	+6.1	0.051	5.871 ± 1.10	+15.7	0.050
		HC	0 M	47	0.271 ± 0.030			8.988 ± 1.02			11.581 ± 1.08			7.742 ± 1.01		
		AD	0 M	27	0.274 ± 0.038	+0.2	0.697	10.404 ± 1.33	+14.8	**<0.001**	12.810 ± 1.40	+9.5	**<0.001**	8.933 ± 1.39	+13.4	**<0.001**
		AD	12 M	27	0.251 ± 0.031	−7.3	**0.009**	10.664 ± 1.41	+18.6	**<0.001**	13.257 ± 1.39	+14.5	**<0.001**	9.367 ± 1.44	+21.0	**<0.001**
Remote	PLIC	HC	0 M	27	0.604 ± 0.039			8.350 ± 0.24			14.928 ± 0.67			5.063 ± 0.35		
functional tracts		mTBI	0 M	24	0.618 ± 0.046	+1.7	0.223	8.644 ± 1.12	+1.6	0.190	15.563 ± 1.99	+2.3	0.125	5.183 ± 0.76	+0.5	0.463
distorted by		mTBI	6 M	24	0.609 ± 0.032	+0.8	0.625	8.403 ± 0.24	+0.6	0.444	15.087 ± 0.45	+1.1	0.332	5.061 ± 0.34	0.0	0.983
ventricles		HC	0 M	9	0.713 ± 0.064			5.350 ± 0.30			9.677 ± 3.40			2.621 ± 0.39		
superior-		NPH Pre-op	0 M	16	0.751 ± 0.034	+5.3	0.063	5.879 ± 0.44	+9.9	**0.004**	12.377 ± 1.01	+27.9	**0.006**	3.255 ± 2.01	+24.2	0.392
inferior		NPH Post-op	6 M	16	0.665 ± 0.077	−6.7	0.122	5.937 ± 0.54	+11.0	**0.007**	11.350 ± 0.99	+17.3	0.075	3.964 ± 2.13	+51.2	0.094
e.g., PLIC		HC	0 M	47	0.525 ± 0.033			7.182 ± 0.47			11.950 ± 0.62			4.791 ± 0.46		
CST		AD	0 M	27	0.518 ± 0.032	−0.4	0.415	7.524 ± 0.45	+3.3	**0.003**	12.437 ± 0.73	+3.1	**0.003**	5.075 ± 0.49	+3.6	**0.014**
		AD	12 M	27	0.517 ± 0.035	−1.5	0.346	7.568 ± 0.45	+5.4	**0.001**	12.464 ± 0.56	+4.3	**0.001**	5.120 ± 0.49	+6.9	**0.005**

*ATR, anterior thalamic radiation; BCC, body of the corpus callosum; CGC, cingulum; CST, corticospinal tract; DTI, diffusion tensor imaging; FA, fractional anisotropy; GCC, genu of the corpus callosum; HC, healthy controls; IFO, inferior fronto-occipital fasciculus; ILF, inferior longitudinal fasciculus; L1, axial diffusivity, L2 and 3, radial diffusivity; MD, mean diffusivity; PLIC, posterior limb of the internal capsule; PTR, posterior thalamic radiation; UNC, uncinate fasciculus.*

^*^Percentage differences and p-values given are for Differences between clinical cohorts compared against healthy controls. The bold values represent significant p values at α <0.05.

**Table 3:** Comparison of example DTI region of interest values within clinical cohorts at baseline and at 6 or 12-month follow-up.

**DTI Regions of Interest**	**Example ROI**	**Cohort**	**Time-point**	**n**	**FA**	**% change**	***p*- value**	**MD (10^**−4**^ mm^**2**^/s)**	**% change**	***p*- value**	**L1 (10^**−4**^ mm^**2**^/s)**	**% change**	***p*- value**	**L 2and 3 (10^**−4**^ mm^**2**^/s)**	**% change**	***p*- value**
Midline right-left tracts adjacent to ventricles e.g., BCC, GCC	BCC	mTBI	0 M	24	0.520 ± 0.065	−4.3	**0.022**	9.162 ± 1.07	−2.9	0.246	15.280 ± 2.21	−4.6	0.137	6.104 ± 0.70	−0.8	0.681
			6 M	24	0.498 ± 0.057			8.898 ± 0.32			14.583 ± 0.87			6.055 ± 0.45		
		NPH Pre-op	0 M	16	0.478 ± 0.097	+2.3	0.601	9.348 ± 1.45	−0.2	0.954	14.430 ± 1.50	+0.1	0.959	6.807 ± 1.67	+5.5	0.486
		NPH Post-op	6 M	16	0.490 ± 0.104			9.329 ± 1.26			14.448 ± 1.22			7.181 ± 2.30		
		AD	0 M	27	0.355 ± 0.053	−6.5	**0.006**	14.202 ± 1.58	+7.8	**0.014**	19.434 ± 1.38	+5.4	**0.010**	11.363 ± 1.83	+12.1	**0.013**
			12 M	27	0.332 ± 0.075			15.315 ± 3.30			20.480 ± 2.65			12.733 ± 3.65		
Lateral to ventricles (long or short) anterior-posterior e.g., ILF, ATR tracts, PTR, CGC	ILF/PTR	mTBI	0 M	24	0.423 ± 0.077	−4.1	0.133	8.953 ± 1.08	−1.4	0.604	13.329 ± 2.77	−3.4	0.438	6.765 ± 0.45	+0.5	0.743
			6 M	24	0.405 ± 0.051			8.824 ± 0.32			12.880 ± 0.81			6.796 ± 0.41		
		NPH Pre-op	0 M	16	0.545 ± 0.051	−11.9	**0.002**	7.807 ± 0.69	−0.1	0.981	13.040 ± 1.38	−6.6	**0.037**	5.191 ± 0.55	+14.0	**0.030**
		NPH Post-op	6 M	16	0.480 ± 0.074			7.802 ± 0.62			12.184 ± 1.20			5.919 ± 1.24		
		AD	0 M	27	0.355 ± 0.028	−2.9	**<0.001**	10.054 ± 0.86	+2.4	**0.009**	13.832 ± 0.97	+1.9	0.127	8.055 ± 0.98	+4.2	0.054
			12 M	27	0.345 ± 0.031			10.300 ± 1.08			14.095 ± 1.10			8.400 ± 1.09		
Fronto-temporal, multi-directional tractse.g., IFO, UNC	IFO/ UNC	mTBI	0 M	24	0.444 ± 0.056	−5.5	**0.032**	9.491 ± 1.09	−2.6	0.306	14.473 ± 2.41	−5.0	0.180	7.000 ± 0.56	−0.2	0.914
			6 M	24	0.420 ± 0.034			9.241 ± 0.34			13.749 ± 0.53			6.988 ± 0.41		
		NPH Pre-op	0 M	16	0.403 ± 0.059	−6.5	**0.032**	7.140 ± 0.42	−0.9	0.567	10.402 ± 0.75	−3.1	0.073	5.358 ± 0.74	+9.6	0.148
		NPH Post-op	6 M	16	0.377 ± 0.054			7.072 ± 0.47			10.083 ± 0.73			5.871 ± 1.10		
		AD	0 M	27	0.274 ± 0.038	−8.3	**0.013**	10.404 ± 1.33	+2.5	0.118	12.810 ± 1.40	+3.5	**0.010**	8.933 ± 1.39	+4.9	**0.018**
			12 M	27	0.251 ± 0.031			10.664 ± 1.41			13.257 ± 1.39			9.367 ± 1.44		
Remote functional tracts distorted by ventricles, superior-inferior e.g., PLIC, CST	PLIC	mTBI	0 M	24	0.618 ± 0.046	−1.6	0.234	8.644 ± 1.12	−2.8	0.314	15.563 ± 1.99	−3.1	0.266	5.183 ± 0.76	−2.4	0.424
			6 M	24	0.609 ± 0.032			8.403 ± 0.24			15.087 ± 0.45			5.061 ± 0.34		
		NPH Pre-op	0 M	16	0.751 ± 0.034	−11.5	**<0.001**	5.879 ± 0.44	+1.0	0.559	12.377 ± 1.01	−8.3	**<0.001**	3.255 ± 2.01	+21.8	0.390
		NPH Post-op	6 M	16	0.665 ± 0.077			5.937 ± 0.54			11.350 ± 0.99			3.964 ± 2.13		
		AD	0 M	27	0.518 ± 0.032	−0.3	0.752	7.524 ± 0.45	+0.6	0.469	12.437 ± 0.73	+0.2	0.800	5.075 ± 0.49	+0.9	0.542
			12 M	27	0.517 ± 0.035			7.568 ± 0.45			12.464 ± 0.56			5.120 ± 0.49		

*ATR, anterior thalamic radiation; BCC, body of the corpus callosum; CGC, cingulum; CST, corticospinal tract; DTI, diffusion tensor imaging; FA, fractional anisotropy; GCC, genu of the corpus callosum; IFO, inferior fronto-occipital fasciculus; ILF, inferior longitudinal fasciculus; L1, axial diffusivity, L2 and 3, radial diffusivity; MD, mean diffusivity; PLIC, posterior limb of the internal capsule; PTR, posterior thalamic radiation; UNC, uncinate fasciculus. The bold values represent significant p values at α <0.05*.

### Arrangement of White Matter Injury by DTI Neuroanatomy and Profile Properties

**Figure 7:**
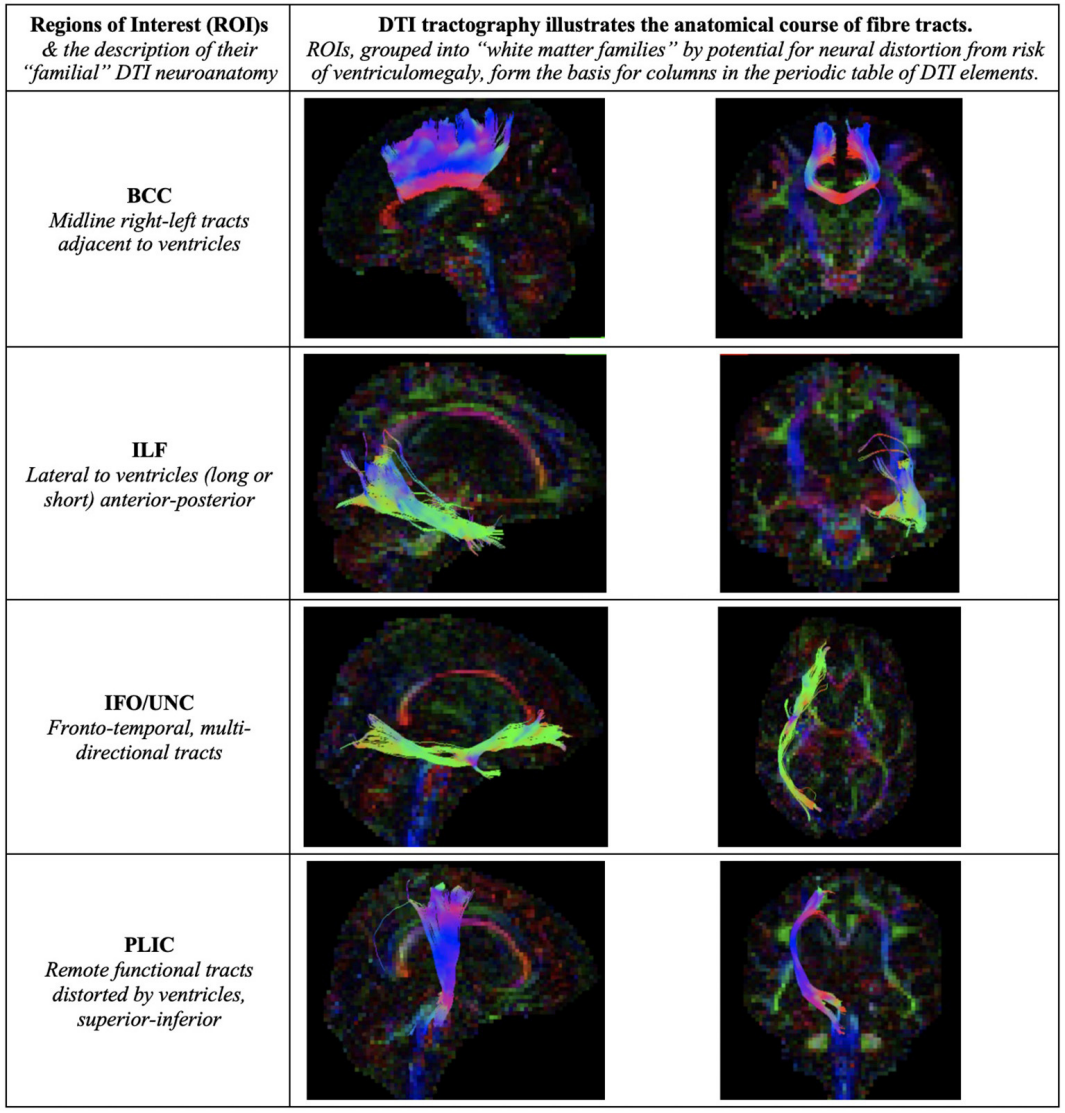
Illustration of Regions of Interest (ROIs). We grouped ROIs into “white matter families” by their predicted potential for neural distortion due to risk of ventriculomegaly. The familial nature of these white matter tracts can be seen in the common anatomical arrangements of their fibers, e.g., right-left or superior-inferior in direction. When grouped in this way, rather than by functional considerations, we demonstrate how tractal families share diffusivity properties. This is the basis for columns in the periodic table of DTI elements. BCC, body of the corpus callosum; ILF, inferior longitudinal fasciculus; IFO/UNC, combination ROI of the inferior fronto-occipital/ uncinate fasciculi; PLIC, posterior limb of internal capsule.

**Figure 8:**
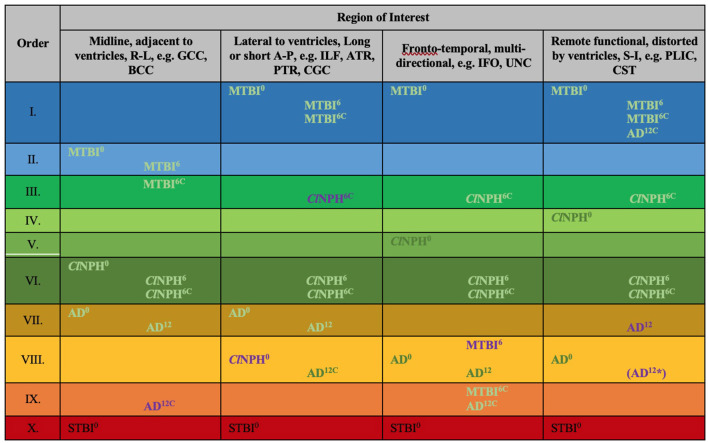
Periodic table of DTI elements. Recurring common properties of DTI profiles arranged vertically by predicted reversibility of white matter injury (see Table 4 and Discussion). ROIs grouped in columns of ‘white matter families' according to distortion potential from ventriculomegaly. Differences at baseline for clinical cohorts at 0 months vs. healthy controls at 0 months mapped onto the periodic table. In addition to baseline DTI profiles, cohorts mapped across timepoints; MTBI^6^, *Cl*NPH^6^ and AD^12^ cohorts are mapped at 6 and 12 months respectively against healthy controls at 0 months; *Cl*NPH^6C^ and MTBI^6C^ are mapped at 6 month follow-up against the respective clinical cohorts at 0 months. Order X has been derived from STBI data in a published cohort by Veenith et al. (77); coincidentally, this unrelated study was performed on the same scanner as the *Cl*NPH cohort being described in this work. ^0/6/12^, DTI profiles at 0 month baseline, 6/12 month follow-up, ^C^, denotes cohorts vs. themselves across timepoints, A-P, anterior-posterior; AD, Alzheimer's Disease; BCC, body of the corpus callosum; *Cl*NPH, Classic NPH; DTI, diffusion tensor imaging; FA, fractional anisotropy; IFO, inferior fronto-occipital fasciculus; ILF, inferior longitudinal fasciculus; L1, axial diffusivity, L2 and 3, radial diffusivity; MD, mean diffusivity; MTBI, mild traumatic brain injury; PLIC, posterior limb of the internal capsule; PTR, posterior thalamic radiation; R-L, right-left; ROI, region of interest; S-I, superior-inferior; STBI, severe traumatic brain injury; UNC, uncinate fasciculus.

We observed recurring common properties in DTI profiles across the spectrum of brain injury and developed a novel arrangement of white matter injury as a “periodic table of DTI elements” (Figure 1, Table 4). We grouped ROIs into columns of “white matter families” according to the distortion potential to their DTI neuroanatomy from risk of ventriculomegaly (Figure 7). We then determined the “Order” of DTI profiles according to predicted reversibility of white matter injury. We reviewed the literature and considered references across multiple neurological conditions of interest that listed the full panel of DTI measures (FA, MD, L1, and L2 and 3), and where pathophysiological findings of disease correlated with DTI interpretation. Following multiple iterations of the initial prototype to accommodate findings from the literature across pathologies, we determined the final hierarchical algorithm for mapping results to the periodic table (Table 5, Figure 8).

**Table 4:** Recurring common properties of DTI profiles, arranged by expected order of white matter reversibility.

**Order**	**Occurrence**	**DTI profile properties**	**White matter injury patterns**	**Key references supporting interpretation of injury**
I.	Difference/ change	No significant difference/ change in cohorts vs. controls or themselves.	Preserved integrity	For diffusion patterns affected by aging, (26, 27).
II.	*Difference/ change*	Small **change** in FA, concurrent and **significant decreases** in MD, L1 and L2 and 3. With further recovery, **increase** in FA due to **significant increase** in L1, **decrease** in L2 and 3.	*Consistent with a range of processes implied by the mechanisms of Neural Repair*	Dynamic changes in DTI profiles in subacute injury appear as DTI conflicts (see Order VIII; at-risk). Recovery is seen with FA increase, significant L1 increase and L2 and 3 decrease (20, 28). In chronic TBI, FA increase, correlated with cognitive functioning, is suggestive of neuroplasticity (29, 30).
III.	*Change only*	**Decrease** in L1 but with $\frac{1}{2}$>2.5-fold (or much higher) **increase** in L2 and 3. A decrease in MD is typical. Decrease in FA due to previous increase in compression, proportional to improvement in L1/L2 and 3.	Improvement in Compression	The hallmark of compression, a predominant increase in L1, is remediable with intervention across hydrocephalic conditions, from acute to chronic (9, 31–33). For this contradictory profile in early and late post-operative stages, Keong et al. (10) and this study. For MD decrease, Ivkovic et al. (34). For a decrease in post-operative FA only in shunt responders, Kanno et al. (19).
IV.	Difference	Driven by **significant** increase in L1, **disproportionate** >2.0-fold vs. MD/L2and3 measures. Increase in FA.	Compression	Typical of acute pediatric hydrocephalus, (31, 32). For NPH, in remote functional white matter families, e.g., PLIC, Hattori et al. (21) and this study. For FA and L1 increases in pediatric hydrocephalus, (35).
V.	Difference	**Increase** in L1 **predominant** (or **predominant decrease** in L2 and 3), **significant** increase in FA/MD.	Stretch/ Compression	For **“predominant stretch/compression”** in NPH/ hydrocephalus, (9, 10, 21, 31, 36). In brain tumors, displacement causes fiber tension or high alignment, Schonberg et al. (15).
VI.	Difference/ change	Driven by L2and3, **highly disproportionate increases** >2.5-fold vs. MD/L1 measures. FA may be **decreased**, even significant	Distortion predominantly due to fluid and /or *post-operative hydrocephalus*	For “**predominant transependymal diffusion with the presence of stretch/compression”** in pre-and post-operative NPH, Keong et al. (10). Increased L2 and 3 and MD reflect axonal disruption, reversal of CSF flow through ependyma and expansion of extracellular space (interstitial oedema); increased L1 is due to stretch. For all 3 changes and decreased FA in pediatric hydrocephalus, Mangano et al. (35). For post-operative young adult hydrocephalus, small ventricles; decreased FA driven by L2 and 3, Tan et al. (37). DTI profile in common for post-operative hydrocephalus; seen across subtypes and white matter tracts.
VII.	Difference	**Disproportionate differences in L 2and 3** >1.5 to <2.5-fold vs. lowest non-FA measure. **Global DTI profile of worsening** **=** concurrent decrease in FA, **increases** in MD, L1, L2 and 3. If L2and3 increase predominant, follow Order VI/VII. If L2and3 increase <1.5-fold or MD/ FA predominant, follow Order VIII/IX.	Oedema and/or loss of integrity	For vasogenic oedema post-TBI, Mac Donald et al. (38) and Veeramuthu et al. (24). For significant decreases in FA due to increases in L2 and 3 in compressive pituitary tumor patients with demyelination, and DTI variability along optic tracts due to anatomy, Paul et al. (39). LI followed by MD increase, were the most sensitive markers in Mild Cognitive Impairment; in MCI and Alzheimer's disease (AD), FA was the least sensitive (25). In early prion disease, Lee et al. (40).
**Order**	**Occurrence**	**DTI profile properties**	**White matter injury patterns**	**Key references supporting interpretation of injury**
VIII.	Difference/ change	**Global DTI profile of worsening**; L2 and 3 increase not disproportionate (<1.5-fold) or predominant/significant individual DTI measure of global change (i.e., FA/MD). **For Differences -MD increase predominant** or**FA decrease predominant** or **FA decrease significant and Global DTI profile;** but fails to match Order VI/VII (not disproportionate increases) or **FA decrease significant and At-risk profile;** decrease in L1, increase in L2 and 3. **For changes –MD increase significant or predominant and global DTI profile; but fails to match order VI/ IX (not disproportionate or too few significant increases)**. **At-risk profile; decrease in L1, increase in L2 and 3**.	*White matter at-risk of injury* disruption due to compression/ stretch/oedemaand/or loss of structure/ atrophy	DTI profile of risk of white matter injury across multiple pathologies. For an NPH model of White Matter At-Risk, Keong et al. (10). For oedema and distension in NPH, (41–45). Stretch component if L1 increase significant. For **“stretch/oedema,”** impact of proximity to ventricle disrupts both axons and periventricular vasculature, causing impaired autoregulation and increased interstitial fluid (10). For a rat model of hydrocephalus, Yuan et al. (46). For ventricular risk/ FA conflicts, (10, 18, 31, 47). For increased MD in pediatric hydrocephalus, reversing with surgery, Isaacs et al. (48). For high self-corrected ΔADC in NPH vs. controls/atrophy, Takatsuji-Nagaso et al. (49). For TBI, Castano-Leon et al. (28) and Sidaros et al. (11); L1 decrease as marker of TBI severity, Lawrence et al. (50). MD increase has been found to be consistently more sensitive than FA decrease in both MCI and AD, across early to late disease (25, 51).
				For Complex vs. Classic NPH; Lock et al. (9). For NPH, MCI and AD; Horinek et al. (52) and Lee et al. (53). For brain tumors, Yuan et al. (54); low anisotropy (q), high isotropy (p), Price et al. (55). For neurodegeneration, (25, 56–64). For decreased FA post-concussion, (12, 30, 65). For vegetative state in ischaemic hypoxic brain injury and TBI, Newcombe et al. (66).
IX.	Change only	FA **decrease significant** & **predominant** or3 **significant concurrent increases** in MD, L1 and L2 and 3. Diffusion in all directions increased with significant loss of microstructural integrity.	Neuronal degeneration	For neurodegeneration, (13, 14, 67–72). For correlation to multimodal MR imaging in atypical AD; Sintini et al. (73). For significant FA reductions, increased with disease duration in prion disease, Lee et al. (40). For axonal degeneration/ demyelination in TBI, Mac Donald et al. (38), Lawrence et al. (50); increases in MD and L2and3. For increases in all 3 non-FA measures in MCI/ AD, Mayo et al. (74) and Bigham et al. (75). In comparing both MCI and AD to controls, effect sizes for MD, L2and3 and L1 were greater than FA; L1 and L2 and 3 increases were the most discriminatory in early vs. late changes respectively (25), Both associated with white matter deficits and clinical impairment; L1 and MD increases being “state-specific,” remaining relatively static with advancing disease, whereas L2and3 increase with FA decrease was “stage-specific,” being increasingly abnormal with disease progression. Longitudinal analysis showed progressive changes in the latter always occurred in areas that had first shown the former; thought to suggest that L1 increase represents an upstream event preceding neuronal loss (51). Despite known limitations of DTI and its variability across scanning sites, consistent findings of early L1 increase and sensitivity of MD over FA as a biomarker of disease reported across AD studies (76). Neurodegenerative changes provide support for DTI profile properties in Orders VII-IX, representing a proposed pattern of progression toward more irreversible injury in the periodic table.
X.	*Difference/ change*	Significant **decrease** in FA, driven by **significant decreases** in MD and L1. L2 and 3 may be either **decreased/ increased/**equivocal.	Swelling/hyper-acute/acute &/or *irreversible injury*	For severe TBI patients, Veenith et al. (77) and Lawrence et al. (50). For worse outcome from chronic TBI, Castano-Leon et al. (28). For reactive astrocytic gliosis without neuronal degeneration in prion disease, Caverzasi et al. (78).

*Mimicking the concept of periods, “Orders” reflect commonly recurring patterns of DTI profiles in white matter (i.e., their “neural” properties) seen in response to injury. Here, we arrange them from Order I to X in our predicted trend from reversible to irreversible brain injury (see Discussion for published literature); interpretation for white matter injury patterns in italics are the authors' proposed hypotheses. In “Occurrence,” we specify whether these “neural” properties are to be found when examining a Difference between cohorts (Patient Cohorts vs. Controls), a Change between them (Cohorts vs. Themselves across different timepoints) or both. To solve the Order of mapping DTI profiles to the periodic table, we devised a hierarchical algorithm of rules (Table 5). The colour coding for the Periodic table signifies the following interpretation of white matter tissue signatures: Blue - Normal-appearing integrity or Healthy DTI profiles. In the Order spectrum from reversible to irreversible DTI profiles, a code akin to a “traffic light” warning system: Green - Potential for reversible brain injury; Amber - Risk of progressing towards irreversibility of injury; Red - Likelihood of irreversible brain injury*.

**Table 5:** Hierarchical algorithm for mapping DTI profiles to the Periodic Table. We mapped Differences between patient cohorts vs. healthy controls at baseline and 6- or 12-month follow-up and Changes between cohorts vs. themselves across timepoints (Tables 2, 3, Figures 3–6). For consistency and reproducibility of interpretation, the Periodic Table requires a Hierarchical Algorithm.

***I. For differences between patients and controls:***				***Algorithm***	***Position***
A1			*No significant changes*. There is a presumption of white matter integrity to avoid *over*-interpretation.	⇒	*Order I*
A2			Contradictory differences, small *change in* FA, significant ↓ MD, L1 and L2 and 3. *In recovery, FA increase, significant ↑ L1 and ↓ L2 and 3*	⇒	*Order II*
A3			DTI profile driven by *significant ↑* L1		
	Bi		Disproportionate ↑ L1>2.0-fold vs. MD/L2 and 3 changes	⇒	*Order IV*
	Bii		L1 ↑ predominant (or predominant ↓ L2 and 3), significant ↑ FA/MD	⇒	*Order V*
A4			DTI profile driven by *significant ↑* L2 and 3		
	Bi		Highly disproportionate ↑ L2 and 3 >2.5-fold vs. MD/L1 changes	⇒	*Order VI*
	Bii		Disproportionate ↑ L2 and 3 >1.5 to <2.5-fold vs. MD/L1 changes	⇒	*Order VII*
A5			Global DTI profile of worsening = ↓ FA ↑ MD ↑ L1 ↑ L2 and 3 *(concurrent) But if -*	⇒	*Order VIII*
	Bi		L2 and 3 ↑ predominant, follow algorithm above, *except if FA/MD highest % value*	*A6Bi and A6Ci*	
			L2 and 3 ↑ <1.5-fold, FA ↓ not significant but MD and L1 ↑ significant ↑ L2 and 3	⇒	*Order VIII*
		Ci	If FA ↓ significant, follow algorithm below	*A4Bi and A4Bii*	
A6	Bi	Ci	Predominant/significant *individual DTI measure of Global change* (i.e., FA/MD) MD ↑ predominant (highest % value) *FA ↓ predominant (highest % value)* FA ↓ significant & a. Global DTI profile *(see A4/A5), disproportionate ↑ L2and3* b. Global DTI profile but not matching Order VI/VII c. At-risk profile: ↓ L1 ↑ L2and3 d. Significant ↓ in MD and L1	⇒ ⇒ ⇒ ⇒ ⇒ ⇒	*Order VIII Order VIII* *Order VI/VII Order VIII Order VIII Order X*
***II. For changes between cohorts and themselves:***				***Algorithm***	***Position***
A1			*No distinct morphological DTI profiles (A2/A4-6) & no significant differences*. There is a presumption of white matter integrity to avoid *over*-interpretation. *Exception for* post-operative hydrocephalus, follow algorithm below and also ⇒	⇒A4Bi	*Order I*
A2	Bi Bii	Ci	Contradictory changes Small ↓ FA, significant ↓ MD, L1 and L2 and 3 *In recovery, FA increase, significant ↑ L1 and ↓ L2 and 3* ↓ L1 but >2.5-fold ↑ L2 and 3 vs. MD/L1 changes *↓ FA significant due to ↑ L2 and 3 with ↓ L1* *↓ L1 significant or predominant (highest % value), ↓ FA significant due to ↓ L1*	⇒ ⇒ ⇒ ⇒	*Order II* *Order II* *Order III* *Order III*
A4	Bi		Highly disproportionate ↑ L2 and 3 >2.5-fold vs. MD/L1 changes Presumed default DTI profile in common for post-operative hydrocephalus	⇒	*Order VI*
A6	Bi	Ci Cii Ciii	MD *↑ significant or predominant (highest % value)* a. Global DTI profile of worsening, but not *A4Bi or A6BiCi-iii*. b. At-risk profile: ↓ L1 ↑ L2 and 3 FA ↓ significant & predominant *(highest % value)* 3 significant ↑ MD ↑ L1 ↑ L2 and 3 Significant ↓ in FA, MD and L1	⇒ ⇒ ⇒ ⇒ ⇒	*Order VIII Order VIII Order IX Order IX Order X*

*To solve the Order of the periods.*

*a. Table 2 for Differences between Patients vs. Controls and. Table 3 for Changes between Cohorts vs. Themselves.*

*b. Resolve the Algorithm in order of (A) Morphological Profiles, (B) Thresholds and (C) Significance of Changes.*

*A. Firstly, describe DTI profiles by their concurrent directions and magnitude of changes (Figures 3–6).*

*What morphological descriptor (A2-A6) best describes the DTI profiles seen? If none match, A1 is the default position.*

*B. Within the morphological descriptor (A2-A6), differentiate DTI profiles by their thresholds for proportions of changes.*

*Disproportion = compare non-FA changes; divide the highest % value by the lowest % value (irrespective of +/- direction).*

*C. Lastly, resolve the remaining DTI profiles by the level of significance of their changes (Tables 2, 3).*

*D. For conflicts that remain unresolved after steps A-C, describe.*

*a. Differences between cohorts by up to two most favorable (lowest ordered) position.*

*b. Changes within cohorts by up to two most favorable (lowest ordered) positions of best fit.*

### Statistical Analysis

Statistical analyses were conducted using SPSS Statistics Version 23.0 (IBM Corp., Armonk, NY, USA). Data is reported as mean ± standard deviation, unless otherwise stated. Independent samples *t*-tests were used to test for differences between clinical cohorts and healthy controls, and paired samples *t*-tests for changes in clinical cohorts over time. All statistical tests were two-tailed, and significance level was set at *p* < 0.05.

## Results

### DTI Profiles Derived at Baseline Across the Spectrum of Brain Injury

At baseline, it was possible to distinguish patient cohorts vs. healthy controls in NPH and AD using DTI profiles alone. When comparing differences in AD patients vs. controls, white matter families in the midline, lateral to the ventricles and remote functional tracts showed evidence of oedema (Figures 3, 4, Tables 2, 4; Order VII). The magnitude of differences was greatest for fronto-temporal tracts, described by Order VIII (white matter at risk) confirming its relevance toward AD pathology.

By contrast, the reverse was true for NPH, where magnitude of changes were worst for white matter tracts in the midline and lateral to the ventricles. The most preserved white matter families were remote functional, followed by fronto-temporal tracts (Figures 3, 4, Tables 2, 4). It was possible to reproduce at least three distinct patterns of white matter injury. The body of the corpus callosum was characterized by Order VI; the inferior longitudinal fasciculus showed worse changes at Order VII; stretch/oedema. By contrast, fronto-temporal and remote functional tracts displayed differences consistent with stretch/compression and compression (Orders V and IV, respectively).

Patients with mTBI displayed white matter tracts that were entirely preserved at baseline compared to healthy controls across all white matter families tested (Figures 3, 4, Tables 2, 4; Order I). By comparison, we further generated DTI profiles from a published dataset of severe TBI (sTBI) patients undergoing normobaric hyperoxia (Veenith et al. (77), an independent study coincidentally performed at the same site as the NPH cohort). We have represented this as Order X due to the life-threatening nature of acute brain injury (Table 4); this does not preclude a change to a more reversible Order with time or following intervention, using the strategy of the *periodic table of DTI elements*. There were no follow-up results presented in this published dataset.

### The Effect of Time and Intervention on DTI Profiles After Brain Injury

Using the cohort with AD from ADNI, we found that it was possible to demonstrate the effect of progression of neurodegeneration. At 12 months' follow-up, the white matter changes progressed in all midline, lateral and fronto-temporal tracts tested (Figures 5, 6, Tables 2–4). When patients were compared to themselves, changes in the midline and fronto-temporal tracts confirmed neuronal degeneration (AD^12C^, Order IX; Figures 5, 6). For tracts lateral to the ventricles, the posterior thalamic radiation showed evidence of loss of integrity/ atrophy (AD^12C^, Order VIII. Only remote functional tracts showed no significant deterioration between cohorts (AD^12C^, Order I), although the cohort at 12 months remained severely disrupted compared to controls at baseline.

In NPH, at 6 months, differences driven by radial diffusivity that were highly disproportionate to other measures remained despite intervention (Order VI). This DTI profile was common to all post-operative white matter families, suggesting that it may be a DTI tissue signature of shunted hydrocephalus. In tracts midline to the ventricles, the pattern of distortion/ oedema (Order VI) was not amenable to improvement. However, white matter families with DTI profiles of stretch/oedema, stretch/compression and compression showed improvement in compression (ClNPH^6C^, Order III).

Interestingly, patients with mTBI, who had uniformly displayed preserved white matter tracts compared to controls at baseline, demonstrated two contrasting new patterns. Changes in fronto-temporal tracts in patients at 6 months' follow-up vs. themselves at baseline were consistent with Order IX, suggesting neuronal degeneration (Figure 6, Tables 3, 4). In the midline tracts, changes evolved at 6 months consistent with hypothesized mechanisms of neural repair (Figure 5, Tables 3, 4; Order II). Figure 8 demonstrates DTI profiles mapped onto the Periodic Table.

## Discussion

In this paper, we examined the utility of creating a simplified taxonomy, to promote transparency and consistency in, and reduce complexity of, interpreting DTI profiles across a spectrum of brain injury. When we grouped white matter tracts by their familial DTI neuroanatomy, rather than their functional organization, we found recurring patterns of DTI measures across cohorts. By deconstructing these morphological patterns into DTI profiles, we were able to observe their periodicity, and so arrange white matter responses according to their diffusivity and neural properties. These properties distinguished white matter injury, when comparing cohorts of mTBI, ClNPH and AD, by the order of their potential reversibility. Using this novel strategy of a *periodic table of DTI elements*, we demonstrated it was possible to characterize cohorts of hydrocephalus vs. non-hydrocephalus and their varying changes over time, and with interventions. The capacity to describe white matter distortion across brain injury cohorts by their neural properties supports a higher diagnostic certainty of neural disruption and more precise evaluation of outcomes from current and emerging therapies. Taken together, the periodic table of DTI elements, and NPH as a human model of reversible disease, show how white matter can recover from varying mechanisms of injury. We term this concept of reversible injury a form of “microstructural resilience.”

At present, such a strategy is lacking; at baseline, cohorts are routinely described by their structural imaging features and less commonly, their white matter metrics, but not by the resilience of their “neural materials.” This is important because reliance on single DTI metrics alone may lead to misleading reports. In literature, fractional anisotropy (FA) is associated with structural integrity, and therefore, a drop in FA is found in most neurological disorders. This reduction in FA would be consistent with microstructural damage of fibers, such as seen in demyelination and axonal degeneration. However, we have found that in hydrocephalus, FA can be shown to both increase and decrease (10, 79). For example, Kanno et al. and our group have reported that a reduction in FA at baseline compared to healthy controls, along with an increase in mean diffusivity (MD), is compatible with cohorts of NPH shunt-responders (10, 18). However, they have also shown a decrease in post-operative FA, only in shunt-responders (19). Whilst this may appear to be an FA conflict, we were able to accommodate both findings, arranged as Order VIII pre-operatively *for “white matter at-risk”* and Order III post-operatively for “*improvement in compression,”* using the periodic table of DTI elements. Indeed, Assaf et al. originally reported both significantly increased and decreased FA in acute pediatric hydrocephalus, with significant decrease in FA (internal capsule) post-surgery (31, 32). Tan et al. also found brain-wide reductions in FA in successfully shunted young hydrocephalic patients (37). Conversely, Hattori et al. and others have also convincingly demonstrated that FA values in NPH patients were higher than in healthy controls (21). Mechanical distortion from ventriculomegaly may increase compaction of fibers along the direction of main diffusivity measures, leading to high FA values. Significant increases in FA or MD may be due to changes driven by predominant increases in L1, even if significant decreases in L2 and 3 are present (9, 10, 21, 31, 35, 36). These DTI profiles of “*compression”* and “*stretch/compression”* appear in the periodic table as neural properties differentiated by proportion; Orders IV and V, respectively.

Apparent DTI conflicts and inconsistencies reflect both the known mathematical derivation of DTI metrics (FA and MD being heavily dependent on changes occurring across other diffusivity measures), as well as specific patterns of white matter tracts and their distortions by CSF compartments described in NPH, such as ventriculomegaly and disproportionately enlarged subarachnoid space hydrocephalus (DESH) (80). Differing brain regions may respond differently to forces of pressure or deformation. The inherent “neural material properties” of white matter affected by pathological processes may determine their capacity for microstructural resilience vs. progressive neuronal degeneration. Differentiation of DTI profiles is important; it allows for multiple patterns of white matter injury to co-exist within individual cohorts. In NPH, we have shown that an *a priori* model of at-risk white matter tracts comprising only six key ROIs is sufficient to demonstrate at least three distinct and concurrent patterns of neural distortion (10). DTI profiles differed according to their risk of proximity to the expanding ventricles. In this study, we have used the strategy of a *periodic table of DTI elements* to reproduce the three known patterns of neural distortion seen in NPH, now refined as Orders IV, V and VI, respectively. We showed that some patterns of injury were more reversible than others. Early post-intervention, we found “*predominant stretch/compression”* to be the most amenable to reversibility; this contradictory decrease in L1 with concurrent increase in L2 and 3, now described as Order III for “*improvement in compression,”* preceded changes in clinical outcome (10). Here, at six months post-shunting, we found “*compression*” and “*stretch/ compression”* to be the most reversible patterns of injury (i.e., pre-operative Orders IV and V, respectively), matching findings from our previous work. Using the periodic table, this profile of improvement is described by Order III. Interestingly, by mapping cohorts to the periodic table of elements, it became apparent that, despite differing pre-operative DTI measures, all post-operative hydrocephalus tracts had a DTI profile in common. Order VI, in which FA decrease is driven by highly disproportionate increases in L2 and 3, was also observed by Tan et al. in their young adult hydrocephalic cohort without ventriculomegaly (37). This suggests Order VI may be a shared tissue DTI signature of treated, post-operative hydrocephalus. In addition, all tracts with improvement shared the DTI profile of Order III.

In this study, we sought to compare DTI profiles in hydrocephalus compared to brain injury and neurodegeneration within the spectrum of reversible to irreversible injury. Using the strategy of a *periodic table of DTI elements*, it has been possible to demonstrate where patterns of neural injury diverge, or where they are overlap. The cohorts in Orders VII to VIII of the periodic table match ADNI studies suggesting that ventriculomegaly may be an early imaging signature of AD and/or NPH. The progression in the AD cohort at 12 months toward neuronal degeneration (Order IX; in midline and fronto-temporal tracts), is consistent with known interpretations of ADNI data; white matter damage in AD follows neurodegenerative staging and progression of disease (75). Despite the known limitations of DTI and its variability across scanning sites, consistent DTI profiles, that of early L1 increase, sensitivity of MD over FA as a biomarker of disease and L2 and 3 increase with FA decrease being increasingly abnormal with disease progression, have been reported across AD studies (25, 51, 76). This provides further support of the morphology of DTI profiles described in Orders VII-IX, representing a proposed progression from reversible to irreversible injury in the arrangement of the periodic table of DTI elements. Our findings also support the model of acute-to-chronic traumatic brain injury with differing patterns of neural response and recovery. In the inferior longitudinal/ uncinate fasciculi, changes at 6 months suggested neuronal degeneration (Order IX). Previous work from a subset of this cohort confirmed that, patients demonstrated cognitive deficits within this timeframe (24). By contrast, at 6 months, DTI profiles seen in the midline tracts (Order II) would be consistent with expected diffusion patterns if pathophysiological processes of neural repair, such as dendritic pruning, influx of inflammatory cells, deposition of biomarker proteins and disorganized neural regeneration, were occurring. Further work is required to characterize these changes, and in particular, confirm that DTI profiles represented by Order II match reports of improvement of cognitive functioning and other deficits in the literature, suggestive of neuroplasticity (29, 30). Presently, the interpretation of DTI profiles is highly dependent upon studies of unambiguous pathophysiological processes, such as stretch distortion/compression due to tumors, variable patterns of oedema (interstitial, vasogenic, cytotoxic), demyelination and Wallerian degeneration [Orders V-IX]. Therefore, in constructing the periodic table, we also sought out further interpretations in literature for comparisons of DTI measures across pediatric to adult hydrocephalus, experimental models of injury, acute-to-chronic TBI, tumors and forms of neurodegeneration (Table 4 for key references), in order to situate DTI profiles within their appropriate context for comparisons.

### Limitations

Our study has a number of limitations. Firstly, ROI methodology has known disadvantages, as compared to more advanced analysis techniques such as semi-automated tractography. However, in our work and that of others, high intraclass correlation coefficients have been reported in clinical use, confirming both its reliability as a research method and applicability to standard practice. Secondly, due to methodological considerations, inter-site DTI variability is a known concern, as diffusivity measures are highly dependent upon machine specifications and acquisition parameters. Whilst this therefore renders it challenging to directly compare DTI output across international collaborative sites, it is still possible to appreciate strong morphological features comprising specific DTI profiles of disease, such as differential diffusion metric sensitivity in early vs. late stages of AD [50, 75; see (Table 4)]. In this study, we have made comparisons across multiple international cohorts but have only compared patients against controls from their own individual sites, thereby avoiding such pitfalls of DTI variability. Furthermore, our findings in normal pressure hydrocephalus are consistent across the variety of techniques employed in literature for DTI post-processing and interpretation (manual to semi-automated methodologies, comprising voxel-based analysis, tract-based spatial statistics, deterministic fiber tractography and ROI-based analyses) (10, 25, 31, 79, 81, 82) confirming the robustness of DTI in its descriptions of microstructural injury.

In reviewing the literature, we included in the analysis sources that listed the full panel of DTI measures, and where pathophysiological findings of disease correlated with DTI interpretation. However, we were limited in that not all papers of interest published their full panel of DTI measures. As global DTI measures are influenced by disproportionate changes in individual metrics, we tried to include papers that took this into account. Additionally, for purposes of clarity, there may be a limit to the number of datasets that can be presented via a periodic table. DTI profiles may be better suited to visual representation in other ways, such as being plotted as a normal distribution curve, or as milestones along a spectrum line from reversible to irreversible brain injury. Subclassifications of DTI profiles may be needed to more accurately represent cohorts with overlapping tissue signatures. Whilst we tested three cohorts vs. controls and one exemplar within a much wider spectrum of brain injury, we expect this methodology to be translatable to other conditions comprising acute-to-chronic pathology. Crucially, the order of potential reversibility of white matter injury is determined *a priori*.

The concept of the periodic table is proposed as a strategy to navigate the pitfalls of DTI interpretation, rather than trying to solve its known shortcomings. This would be the first description of a taxonomy for classifying and describing DTI profiles, and we expect that this will only be its first iteration. We shall seek to address the limitations and considerations mentioned in future work; we hope that community efforts may contribute toward this concept.

## Conclusion

Our study has demonstrated that it is possible to distinguish between different cohorts along the spectrum of brain injury by describing the properties of their DTI white matter profiles. We proposed a simplified taxonomy to improve the reproducibility and reduce the complexity of DTI interpretation to differentiate cohorts of hydrocephalus vs. non-hydrocephalus using a novel strategy of a periodic table of DTI elements. In this way, DTI profiles provide, both at baseline and in response to interventions, a form of rapid characterization of cohorts within the context of known tissue signatures across pathologies. As potential reversibility of white matter injury is assessed *in vivo*, this methodology may contribute to the understanding of the use of biomarkers to track changes in disease cohorts.

## Data Availability Statement

The raw data supporting the conclusions of this article will be made available by the authors upon reasonable request.

## Ethics Statement

The studies involving human participants were reviewed and approved by local or institutional Ethics Committees. Written informed consent was obtained from all subjects or legal representatives, if appropriate, as required by local Ethics Committees. The mTBI study was regulated by the University of Malaya Research Ethics Committee and Hospital Ethics Committee (UM/EC Ref: 947.15). The NPH study was approved by the local Research Ethics Committee of the Cambridge Health Authority (LREC: 06/Q0108/330). Individual ADNI sites received approval from their respective governing Institutional Review Boards. The patients/participants provided their written informed consent to participate in this study.

## Author Contributions

NK conceptualized the experimental design. NK, CL, and SS analyzed the data. NK and CL wrote the manuscript, as well as provided visualizations for the data and led revisions of the work. AH contributed to data acquisition, imaging post-processing, and analysis from the UM site. ZC, MC, JP, and VN contributed to the acquisition of data used in this paper and provided comments and suggestions toward improving revisions. All authors reviewed the manuscript.

## Funding

NK was supported by a National Medical Research Council Clinician Scientist Award (MOH-000303-00) and Transition Award (NMRC/TA/0024/2013) and the National Neuroscience Institute RIE2020 Centre Grant Bridging Fund (IRNMR17CBG01). NPH study imaging at the University of Cambridge was funded by a Medical Research Council Programme Grant [Wolfson Brain Imaging Centre Cooperative]. MC and ZC were supported by Revert Project, Interreg France (Channel Manche) England, funded by ERDF. JP has received a National Institute for Health Research Brain Injury Health Technology Cooperative and National Institute for Health Research Senior Investigator Award (2009–2014). MTBI data from the University of Malaya was partially funded by a University Malaya Research Grant (UMRG; RG008C-13HTM) and a High Impact Research Grant of University of Malaya (HIR-UM.C/625/1/HIRMOHE/12).

## Conflict of Interest

The authors declare that the research was conducted in the absence of any commercial or financial relationships that could be construed as a potential conflict of interest.

## Publisher's Note

All claims expressed in this article are solely those of the authors and do not necessarily represent those of their affiliated organizations, or those of the publisher, the editors and the reviewers. Any product that may be evaluated in this article, or claim that may be made by its manufacturer, is not guaranteed or endorsed by the publisher.
